# Delayed diagnosis and treatment of achalasia: a case report

**DOI:** 10.1186/s13256-024-04717-7

**Published:** 2024-10-15

**Authors:** Sabrina Ginsburg, Chelsea Caplan, Gauri Agarwal

**Affiliations:** University of Miami Leonard M. Miller School of Medicine, Miami, USA

**Keywords:** Achalasia, Dysphagia, Esophagram, Heller myotomy, Case report

## Abstract

**Background:**

Achalasia is characterized as an esophageal motility disorder with incomplete relaxation of the lower esophageal sphincter. Achalasia can be associated with abnormal peristalsis and symptoms of dysphagia, acid reflux, and chest pain. The exact pathophysiology of achalasia remains unclear, but it is hypothesized to be due to degeneration of the myenteric plexus.

**Case presentation:**

In this case, a 46-year-old Hispanic man presented to the emergency room with a 12-year history of progressive discomfort with swallowing solids and liquids. Due to many years of incomplete follow-up care and lack of understanding of the course of his disease, this patient’s symptoms escalated to complete intolerance of oral intake and significant weight loss. He was diagnosed with achalasia during his hospital stay and treated successfully with laparoscopic Heller myotomy.

**Conclusions:**

This case discussion illustrates the importance of follow-up care and patient education so that diagnosis and treatment of achalasia are not delayed.

## Background

Achalasia is a rare condition that causes esophageal motor dysfunction due to lack of relaxation in the lower esophageal sphincter (LES). The incidence of achalasia varies throughout the world, but the approximate worldwide annual incidence is 1/100,000 [1, 2]. The condition typically presents with symptoms of dysphagia with solids that progresses to an intolerance of liquids. Nonspecific symptoms of regurgitation along with chest pain, cough, and weight loss are common [1, 3]. Due to dysphagia and other symptoms, patients often adopt certain habits such as eating more slowly and avoiding meals in public settings. These techniques can slow the progression of the discomfort a patient may experience and can delay a need to seek medical attention [4].

The pathophysiology of achalasia is not well defined but is thought to be related to the degeneration of the myenteric plexus, a network of nerve cells between the layers of esophageal muscles, which are responsible for peristalsis and relaxation of the lower esophageal sphincter. There have been associations between achalasia and parasitic infections (*Trypanosoma cruzi*), viral infections (*Herpes simplex virus*), malignancies (esophageal), as well as autoimmune disorders such as systemic lupus erythematosus [5]. Approximately 7–10% of individuals with achalasia are infected with *T. cruzi*, also known as Chagas disease [6].

There are various treatment approaches for achalasia including endoscopic treatment via balloon dilatation, botulinum toxin, laparoscopic Heller myotomy, and esophagectomy [7]. A meta-analysis performed by Schoenberg *et al*. concluded that laparoscopic Heller myotomy should be considered first-line treatment of achalasia as it demonstrates greater efficacy than balloon dilatation, but ultimately the treatment depends upon the patient’s suitability for surgery [4, 8].

## Case presentation

A 46-year-old Hispanic man presented to the emergency department reporting a 12-year history of discomfort when swallowing solids and liquids, which had acutely worsened over 2 weeks. He described a “tight” sensation in his chest any time he ingested food or drink and stated that the contents were getting stuck in his throat. He could no longer eat foods larger than half an inch and would have postprandial vomiting with any larger substance. The patient felt that his voice was becoming progressively hoarse and he was experiencing occasional symptoms of reflux. He reported that he had lost approximately 10 lbs over the past 2 months because of these symptoms, and a total of 55 lbs since the onset of his symptoms 12 years prior (starting weight: 220 lbs, weight on admission: 165 lbs). He denied odynophagia or recent fevers, chills, headaches, lightheadedness, cough, shortness of breath, chest pain, back pain, abdominal pain, diarrhea, constipation, or dysuria. His past medical history was significant for hypertension, for which he was taking amlodipine, and he was taking no other medications. He had a previous history of appendectomy but otherwise denied any abdominal surgeries. He was born in Mexico, worked as a landscaper, and was an active smoker with a 30-pack year history. He denied any alcohol or illicit drug use, and denied any family history of gastrointestinal disorders or malignancies.

The patient had visited the emergency room at other hospitals multiple times over the past few years for similar symptoms without a definitive diagnosis or treatment. On presentation to our hospital, his initial vital signs were unremarkable. His laboratory results showed a white blood cell count of 15.2/µL, hemoglobin of 12.5 g/dL, and a platelet count of 178,000/µL. Electrolytes, creatinine, and liver enzymes were all within normal limits. The patient was administered 1 L of normal saline and metoclopramide for nausea.

A chest x-ray revealed questionable gaseous distention of the esophagus and a computed tomography (CT) of the chest/neck was performed for further evaluation. The CT revealed a significantly distended esophagus with distal tapering at the gastroesophageal (GE) junction and stenosis of the lower esophageal sphincter, suggestive of achalasia without a mass lesion. The CT visualized associated fluid and food contents putting this patient at risk for aspiration. These findings were concerning for structural or functional dysmotility.

**Fig. 1:**
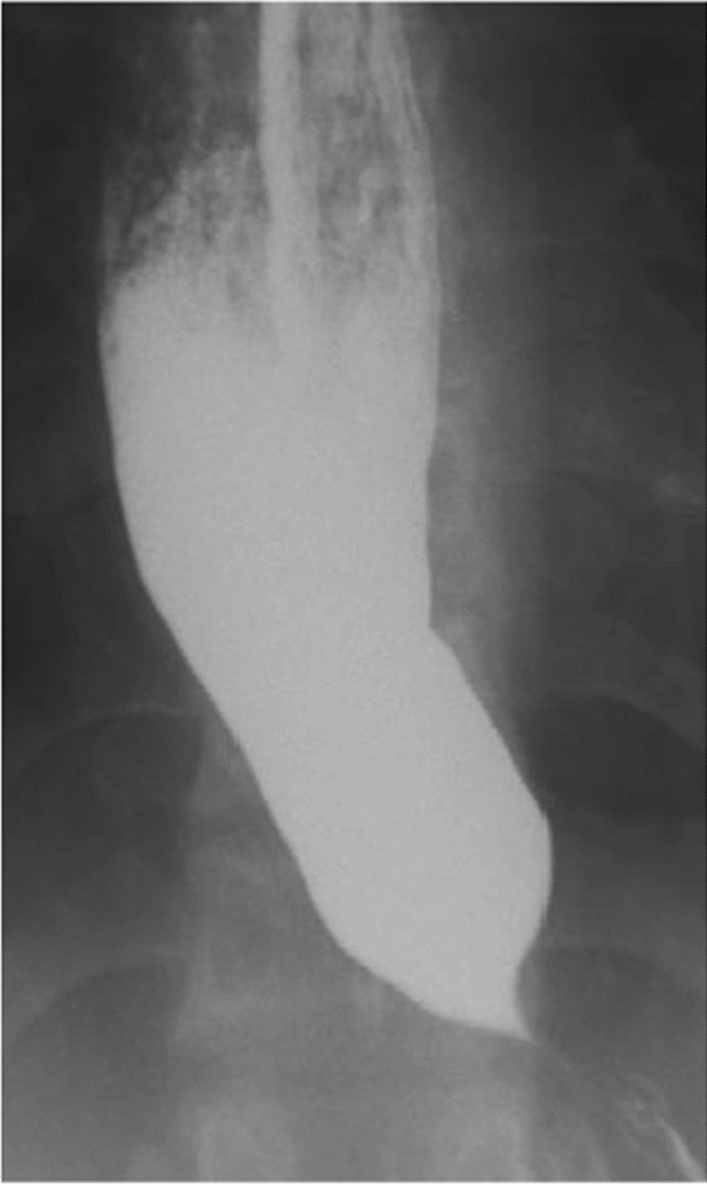
Bird-beak deformity of distal esophagus in a patient with achalasia. Reproduced with permission from: Xiang et al. [9]

During hospitalization, an esophagram confirmed achalasia with a classic “bird-beak” configuration. Figure 1 represents an example of a dilated upper esophagus with the tapered narrowing of the distal esophagus commonly referred to as the bird-beak deformity [9]. Pathology from esophageal biopsies revealed *Candida* (likely due to stasis from dysmotility) and treatment with intravenous fluconazole was initiated. Peripheral blood smear ruled out Chagas disease. On day 8 of his hospitalization, he was taken to the operating room (OR) for laparoscopic Heller myotomy with no complications. On day 11, a follow-up esophagram was completed and revealed normal findings with mild ballooning of the distal esophagus. The patient was discharged on day 12 after the patient was tolerating a bariatric stage 1 diet. He remained hemodynamically stable throughout his hospital stay. The patient visited the clinic 4 days after discharge and was able to tolerate a clear liquid diet. He denied nausea, vomiting, hematemesis, reflux, or bloody stools. A total of 1 week after discharge, he was able to tolerate a regular diet.

## Discussion

The emergency department (ED) focuses on stabilizing and immediately ruling out life threatening illnesses. Many of the patients who are stable and have no urgent need for hospital admission are discharged and encouraged to follow up with a primary care provider. The rising wait time, lack of accessible clinics, and insurance barriers make the transition from the ED to a primary care physician (PCP) outpatient appointment anything but seamless. In a recent study led by Yale University, it was shown that only 26% of people from this sample who were discharged from the ED were able to get a PCP appointment within a week [10].

In this case report, the patient went to the ED multiple times with a slight progression in the disease course with each visit, over a period of 12 years. It was not until the final visit, where he was completely solid food intolerant, that a diagnosis of achalasia was determined. Because of its rarity, achalasia is known to have a greater delay from symptom onset to final diagnosis with the average being 4–5 years [1]. Unfortunately for this patient, he was symptomatic for 12 years before receiving his diagnosis and treatment, which is more than double the average time lapse.

Chagas disease, commonly seen in South America, has a known association with achalasia. The esophagopathy seen with Chagas disease is known to have a slow development over many years [7]. No evidence for Chagas disease was found in this patient. Another contributing factor to this patient’s late diagnosis was his low health literacy and the lack of continuity of care. Health literacy is recognized as a stronger predictor of unfavorable health outcomes compared to age, income, employment status, education level, or race [11]. Patients with low health literacy utilize more resources through continued trips to the emergency room and experience more frequent hospitalizations. A systematic review analyzing patients in emergency departments found that a large group of the patients did not have adequate health literacy, which contributes to more frequent ED use and overall, more resources being used [12].

Achalasia is a rare disease with a broad presentation that can be associated with diagnostic delay and misdiagnoses. There are few published articles about the recognized delay in diagnosis for achalasia compared with other conditions. Although not understood, atypical symptoms on presentation, misleading descriptions, and misinterpretations of diagnostic studies seem to be the leading variables. A study by Muller *et al*. compared these variables with 300 patients recently diagnosed with achalasia. The results showed that the presentation of “heartburn” or “nausea” was associated with a higher rate of delay in diagnosis [13]. Medical professionals should consider achalasia in patients with such symptoms. Inflammation of the esophageal mucosa after years of food impaction can cause retention esophagitis, which results in an increased risk for esophageal cancer [14].

## Conclusion

The diagnosis of achalasia, a rare disorder causing esophageal dysmotility, can be delayed several years for a number of reasons including insidious onset, nonspecific symptoms, and low health literacy. To improve quality of life in patients with achalasia and prevent long term complications, it must remain on the differential for dysphagia so that the length of time between symptom onset to treatment is decreased. This will require vigilant screening in PCP and ED settings.

## Data Availability

Not applicable.

## Abbreviations

LES: Lower esophageal sphincter
ED: Emergency department
PCP: Primary care physician
